# Whole genome sequences of nine *Taylorella equigenitalis* strains isolated in the Czech Republic between 1982–2021: Molecular dating suggests a common ancestor at the time of Roman Empire

**DOI:** 10.1371/journal.pone.0315946

**Published:** 2025-01-03

**Authors:** Matěj Hrala, Petr Andrla, Juraj Bosák, Pavla Fedrová, Amir Mugutdinov, Renata Karpíšková, Kateřina Nedbalcová, Jitka Raichová, Martin Faldyna, Petr Hořín, David Šmajs

**Affiliations:** 1 Department of Biology, Faculty of Medicine, Masaryk University, Brno, Czech Republic; 2 Department of Public Health, Faculty of Medicine, Masaryk University, Brno, Czech Republic; 3 Department of Infectious Diseases and Preventive Medicine, Veterinary Research Institute, Brno, Czech Republic; 4 National Stud at Kladruby nad Labem, Kladruby nad Labem, Czech Republic; 5 Department of Animal Genetics, Faculty of Veterinary Medicine, University of Veterinary Sciences Brno (VETUNI), Brno, Czech Republic; 6 Institute of Medical Genetics and Genomics, Faculty of Medicine, Masaryk University, Brno, Czech Republic; North Carolina State University, UNITED STATES OF AMERICA

## Abstract

*Taylorella equigenitalis* is the causative agent of sexually transmitted contagious equine metritis. Infections manifest as cervicitis, vaginitis and endometritis and cause temporary infertility and miscarriages of mares. While previous studies have analyzed this organism for various parameters, the evolutionary dynamics of this pathogen, including the emergence of antibiotic resistance, remains unresolved. The aim of this study was to isolate contemporary strains, determine their genome sequences, evaluate their antibiotic resistance and compare them with other strains. We determined nine complete whole genome sequences of *T*. *equigenitalis* strains, mainly from samples collected from Kladruber horses in the Czech Republic. While *T*. *equigenitalis* strains from Kladruby isolated between 1982 and 2018 were inhibited by streptomycin, contemporary strains were found to be resistant to streptomycin, suggesting the recent emergence of this mutation. In addition, we used the collection dates of Kladruber horse strains to estimate the genome substitution rate, which resulted in a scaled mean evolutionary rate of 6.9×10^−7^ substitutions per site per year. Analysis with other available *T*. *equigenitalis* genome sequences (n = 18) revealed similarity of the Czech *T*. *equigenitalis* genomes with the Austrian *T*. *equigenitalis* genome, and molecular dating suggested a common ancestor of all analyzed *T*. *equigenitalis* strains from 1.5–2.6 thousand years ago, dating to the first centuries A.D. Our study revealed a recently emerged streptomycin resistance in *T*. *equigenitalis* strains from Kladruber horses, emphasizing the need for antibiotic surveillance and alternative treatments. Additionally, our findings provided insights into the pathogen’s evolution rate, which is important for understanding its evolution and preparing preventive strategies.

## Introduction

*Taylorella equigenitalis* is the causative agent of sexually transmitted contagious equine metritis (CEM). Infected mares may exhibit a range of clinical signs, including abundant mucopurulent vaginal discharge, vaginitis, endometritis, and cervicitis. However, the severity of these signs varies considerably among individual animals. Notably, some infected mares and all infected stallions remain asymptomatic [1, 2]. As the acute form of infection leads to temporary infertility in mares, this infection causes substantial economic losses. The antibiotic resistance profiles of *T*. *equigenitalis* strains remained unchanged over time. Streptomycin resistance was the only variable resistance, reflecting the relatively low genetic diversity of these strains [3, 4].

Molecular typing using gel electrophoresis for epidemiological tracing of *T*. *equigenitalis* provided limited insight into clonality, as most strains tested showed little or no variability [5, 6]. Multilocus sequence typing (MLST) provides a more detailed view of the genetic diversity of *T*. *equigenitalis* by analyzing seven housekeeping genes and is well suited for large-scale epidemiological studies [7]. A previous comprehensive study using MLST compared 367 *T*. *equigenitalis* strains and revealed a total of 49 sequence types (STs) belonging to three major and several minor clusters [8]. Despite the fact that *T*. *equigenitalis* strains were isolated in more than a dozen different countries over a period of four decades, relatively low genetic heterogeneity was found among the *T*. *equigenitalis* strains tested (0.13 STs/strain; [8]). Czech strains (from Kladruber horses) clustered together with German and Swiss strains, whereas Austrian strains (from Lipizzaner horses) clustered separately. The first complete whole genome sequence of *T*. *equigenitalis* (MCE9 strain, isolated from a stallion in Haute-Savoie, France) was published in 2011 [9], followed by the genome sequence of its close relative, *T*. *asinigenitalis*, responsible for similar infections in donkeys, in 2012 [10]. Since then, several studies have determined the whole genome sequences of additional *T*. *equigenitalis* strains, including those by Hauser et al. [11], Hébert et al. [12], May et al. [13], Hicks et al. [4], and Melzer et al. [14]. However, most of the available *T*. *equigenitalis* genome sequences are draft genome sequences. While most of the previously sequenced genomes were generated using 454, Ion Torrent or Illumina sequencing, this study employed a hybrid approach, combining long-read MinION sequencing used for scaffolding with short-read Illumina sequencing used for deep sequencing coverage.

To elucidate the genetic diversity of *T*. *equigenitalis* and advance molecular typing methodologies, a whole-genome sequencing approach encompassing multiple geographic regions and time points is required. Significant genetic diversity and limited evidence of recombination has been observed among strains introduced into the USA, with this diversity primarily driven by variations in repeat-containing sequences and specific genomic regions [4]. However, knowledge gaps regarding the evolutionary dynamics of this pathogen remain.

While previous studies have provided valuable insights into the genetic diversity of this bacterium, further studies providing a deeper understanding of its evolutionary dynamics and the emergence of antibiotic resistance are crucial for effective disease management and prevention. The aim of this study was to isolate contemporary *T*. *equigenitalis* strains from Kladruber horses in the Czech Republic, determine their antibiotic susceptibility, determine their genome sequences and compare them with other available sequenced strains. Moreover, the genome sequences were also used to elucidate the clonality and evolutionary history of *T*. *equigenitalis*. The isolated breeding of Kladruber horses, which prevented external factors from influencing the evolutionary trajectory of the pathogen, served as a convenient source of *T*. *equigenitalis* strains for the study of evolutionary dynamics.

## Material and methods

### Sample collection and identification of *T*. *equigenitalis* isolates

All of the 33 Kladruber stallions tested in this study originated from two national stud farms in Kladruby nad Labem (n = 24) and Slatiňany (n = 9), Czech Republic, and were sampled between the years 2021 and 2023. Samples (n = 108) were taken from preputium, *fossa glandis* and urethra of stallions using a sterile cotton swab. In some cases (n = 16), ejaculatory samples were also collected and used for screening. All samples were collected during mandatory routine veterinary screenings for *T*. *equigenitalis*. Samples were submerged in Amies transport medium in labelled tubes, refrigerated and transported to the laboratory. Within 24 to 48 hours, samples were streaked on chocolate agar plates supplemented with NAD (nicotinamide adenine dinucleotide) and hemin (LabMediaServis, Czech Republic). To minimise the growth of commensal microflora, agar plates were supplemented with 5 μg·ml^-1^ amphotericin B and clindamycin (Glentham Life Sciences, United Kingdom). The plates were incubated for 4 days at 37°C in Brewer anaerobic jar with a pre-mixed gas (5% CO_2_, 1.5% O_2_, balance N_2_) supplied from a cylinder (Messer TechnoGas, Brno, Czech Republic). After 4 days, the plates were inspected and photographed, and suspect colonies (i.e., small and transparent colonies; S1 Fig) were isolated and re-streaked to obtain a pure *T*. *equigenitalis* culture. Isolated strains were stored in the laboratory as cryogenic stocks.

To confirm *T*. *equigenitalis*, two loci (*glt*A and *txn*), previously described in the *T*. *equigenitalis* MLST system [7], were PCR amplified. The following primers were used: glta-F, 5’-GCTCAGACAGGCATGTTTACTTA-3’; glta-R, 5’-GTCCCCATAGGCAAGAAATAC-3’; txn-F, 5’-ACGGGGGACCGCATAAAGCC-3’; txn-R, 5’-AGCGTTTCTGTACCCGTGCGA-3’, resulting in amplicons of 682 and 455 bp, respectively. Each reaction contained 0.2 μl of each primer (100 pmol/μl), 0.4 μl of a 10 mM deoxynucleotide triphosphate (dNTP) mixture, 2 μl of ThermoPol Reaction buffer, and 0.08 μl of *Taq* polymerase (5000 U/ml; New England BioLabs, Ipswich, MA, USA). For each reaction, 1 μl of bacteria in water (one bacterial colony resuspended in 200 μl of distilled water) was used as template. The reaction mixture was supplemented with PCR grade water to a final volume of 20 μl. Negative controls containing no DNA were included. PCR amplification was performed under the following cycling conditions: 94°C (5 min); 94°C (30 s), 55°C (30 s) and 72°C (1 min) for 35 cycles; and 72°C (7 min). PCR products were visualised on agarose gels and, in the case of *gltA*, positive samples were Sanger sequenced at Eurofins Genomics Company (Ebersberg, Germany; S1 Fig). NCBI BLAST search was used to confirm the identification of *T*. *equigenitalis* sequences, and search results with ≥99% identity and ≥95% query coverage were considered positive. The sequencing identification codes of individual reads are shown in S1 Fig and reads are available on request.

Four additional historical *T*. *equigenitalis* strains of Czech origin were included in the whole genome analysis. These strains included CAPM 6344 and CAPM 6345, isolated in Kladruby nad Labem in 1982, and strains CAPM 6606 and CAPM 6629, isolated in Kyjov and Tlumačov in 2017 and 2018, respectively (provided by the Veterinary Research Institute, Brno, Czech Republic). The strain CAPM 6629 (Tlumačov; 2018) was isolated from a different breed of horse (unknown breed) than the Kladrubers, but the horse had regular contact with Kladruber horses.

In addition, the *T*. *equigenitalis* reference strain CCM 6190^T^ (labelled as UK1 in the analysis), originally isolated in England in 1977, was obtained from the Czech Collection of Microorganisms (Brno, Czech Republic). In addition, the assembly of the same sample, completed by the Wellcome Sanger Institute, was retrieved from NCBI under assembly number 48853_G02. This assembly was then included in the analysis as sample UK2. Four distinct single nucleotide variants (SNVs) were found between the genome sequences of UK1 and UK2.

### DNA isolation and whole-genome sequencing

A combination of MinION nanopore and Illumina sequencing was used to determine the complete genome sequences.

For MinION nanopore sequencing, DNA extraction was performed using the DNA-MagAttract HMW DNA Kit (Qiagen, Hilden, Germany). The library for Nanopore sequencing (Oxford Nanopore Technologies, Oxford, UK) was prepared using a ligation kit SQK-LSK109 with native barcoding (EXP-NBD104) according to the manufacturer´s instructions with the following modifications: i) extended incubation time with beads on Hula mixer to 10 min (instead of 5 min), ii) washing of magnetic beads with 80% ethanol (originally 70%). During the library preparation and barcoding steps, iii) the pellet was resuspended in an elution buffer instead of nuclease-free water, as it offers better DNA stability and minimizes the risk of degradation during the sequencing process, iv) the incubation step was extended to 10 min at 37°C (instead of 2 min at room temperature) to further optimize adapter ligation efficiency. Following library preparation, the pooled libraries were sequenced on a SpotON flowcell (R9.4.1) for 48 hours, using the MinION nanopore sequencer. Basecalling and barcoding were performed using Guppy v6.0.1 [15] with the high accuracy model, employing a minimum quality score threshold of 9 to ensure reliable and accurate basecalling results.

For Illumina library preparation and sequencing, DNA extraction was performed using the QIAamp DNA Blood Midi Kit (Qiagen, Hilden, Germany). DNA samples were used to prepare sequencing libraries (NEBNext DNA Library Prep Kit, NEB), which were sequenced at Novogene (Beijing, China) on an Illumina HiSeq instrument, generating 150 bp paired-end reads. To ensure the highest data quality, quality controls were performed using tools such as FastP [16], FastQC [17], MultiQC [18], and FastQ Screen [19]. FastQC and FastP assessed overall data quality, while FastQ Screen identified potential contamination and MultiQC aggregated quality control results.

### *De novo* genome assembly

Sequencing reads from both Illumina (short reads) and MinION nanopore (long reads) platforms were subjected to *de novo* assembly using Unicycler v0.5.0 [20] with default parameters, employing a hybrid mode to maximize the benefits of both read types. We used Samtools 1.14 [21] to exclude unmapped or poor quality reads, secondary alignments and improperly paired reads. To assess the quality of the resulting assemblies, coverage was calculated using the DepthOfCoverage function from GATK v3.7 [22] for Illumina reads and Samtools depth for nanopore reads. The mean coverage was determined to be within the range of 495× to 700× for Illumina reads and 237× to 606× for Nanopore reads. Following assembly, a thorough manual inspection of the assemblies was performed using the Integrative Genomics Viewer (IGV) 2.4.9 [23] and sequence analysis software (SeqMan Pro®. Version 10.1.1. DNASTAR. Madison, WI).

### Antibiotic susceptibility testing

The susceptibility of all 10 sequenced strains to streptomycin, gentamicin, nitrofurantoin, rifampicin, and sulfamethoxazole/trimethoprim was evaluated using the disk diffusion method. Briefly, each bacterial isolate was spread on a chocolate agar plate using a sterile microbiological loop. Antibiotic disks containing streptomycin (10 μg), gentamicin (10 μg), nitrofurantoin (100 μg), rifampicin (5 μg) and sulfamethoxazole/trimethoprim (25 μg) (Oxoid, Basingstoke, UK) were placed on the inoculated agar surface. Plates were then incubated for 48 hours at 37°C in a 5% CO_2_ and 1.5% O_2_ atmosphere. The diameters of the zones of growth inhibition surrounding each disk were measured using vernier caliper.

### Global dataset of publicly available genome sequences

Eighteen publicly available *T*. *equigenitalis* genomes were retrieved from the NCBI GenBank repository (February 2023). In this dataset, information was obtained on the majority of samples, including their respective collection dates. The dataset included strains from 8 countries, collected in Europe (five), South Africa, South Korea and the United Arab Emirates (S1 Table). The reference genome of *T*. *asinigenitalis* (ASM22662v1), a related species, was also retrieved from the NCBI GenBank.

### Identification of potential recombination sites in *T*. *equigenitalis* genomes

Multiple sequence alignment and variant calling was performed using BactSNP v1.1.0 [24], with a reference genome of *T*. *equigenitalis* MCE9 (GenBank GCA_028868935.1). The aligned genomes were run through Gubbins v3.3.0 [25] using a Hasegawa, Kishino and Yano (HKY) nucleotide substitution model [26] to identify putative recombination sites (S2 Table). These sites were masked in the alignment using the Gubbins tool, mask_gubbins_aln.py.

### Model selection

The optimal model was selected using nested sampling. To evaluate the suitability of either the strict clock, which assumes a constant rate of evolution across all lineages, or the uncorrelated relaxed clock, which allows for variation in evolutionary rates between lineages, for our dataset, initial models were generated using tip dates, including the HKY substitution model and the coalescent constant population model. Both models underwent assessment using the Nested Sampling Bayesian computational algorithm v1.1.0 [27] within the BEAST2 package, employing a particle count of 1 and a subchain length of 5,000. This analysis provided a compelling support for the strict clock model. Various population models were compared to ensure the identification of the most appropriate one. These encompassed the Coalescent Constant Population, the Birth Death model, and the Yule Skyline model. This analysis identified the Coalescent Constant Population as the optimal tree model (S3 Table).

### Evolutionary rate estimation

The final recombination-free alignment was used to reconstruct the phylogeny using the Bayesian framework implemented in BEAST v2.7.4 [28]. Strains from Kladruby, Kyjov, and Tlumačov (all from the Czech Republic and all from Kladruber horses, except for the strain from Tlumačov, which originated from a different horse breed) were used to estimate the clock rate. Tip dates were specified based on the basis of the years of sample collection. The HKY model was employed, and substitution rates were estimated using a normal distribution in BEAST. To infer evolutionary history, the strict clock model was used in combination with the coalescent constant population demographic model. The BEAST analysis was run for 10,000,000 Markov Chain Monte Carlo (MCMC) generations with a 10% burn-in, as these initial iterations may not accurately reflect the true distribution of parameters. A total of 5 runs were combined using LogCombiner 2.4 [28], resulting in a total of 45 million generations. Parameter estimates were obtained using Tracer v1.7.2 [29], and a maximum clade credibility tree was generated using TreeAnnotator v2.7.4 [28]. The resulting tree was visualised using FigTree v1.4.4 [30]. The scaled mean evolutionary rate for the whole genome was determined to be 6.8511×10^−7^ substitutions per site per year (95% HPD; highest posterior density: 4.8564×10^−7^–8.6941×10^−7^; this interval is the range of values within which the true evolutionary rate is estimated to lie with 95% confidence; S4 Table).

### Phylogenetic analyses

All measured and downloaded genome sequences were included in this phase of the analysis using BEAST v2.7.4. Again, tip dates were specified based on the years of sample collection, approximating the year 2000 when precise information was lacking. The HKY85 model panel, the coalescent constant population demographic model and the strict clock model were employed in this phase as well. However, the substitution rates were fixed at the previously calculated value of 6.8511×10^−7^. The extended BEAST analysis was run for 10,000,000 MCMC generations with a 10% burn-in. Combining 5 runs through LogCombiner 2.4 [28], a total of 45 million generations was obtained. Parameter estimates were calculated using Tracer v1.7.2 [29], and a maximum clade credibility tree was generated using TreeAnnotator v2.7.4 [28]. The resulting tree was visualized using FigTree v1.4.4 [30].

### Data availability

The raw sequencing reads from Illumina were uploaded under the BioProjects No. PRJNA1111427, PRJNA1111417, PRJNA1111418, PRJNA1110976, PRJNA1111292, PRJNA1111304, PRJNA1111331, PRJNA1111353, PRJNA1111378, and PRJNA1111394. S5 Table summarises the GenBank SRA and complete genome sequence accession numbers.

## Results

### Isolation of *T*. *equigenitalis* from Kladruber stallions

Out of 33 Kladruber stallions tested, 10 stallions (30.3%) tested positive for *T*. *equigenitalis* cultivation (Table 1). *T*. *equigenitalis* was preferentially isolated from the *fossa glandis* (9 positive out of 10 tested), the *ostium urethrae* (4 positive out of 10 tested), the *praeputium* (0 positive out of 10 tested) and the ejaculate (2 positive out of 4 tested). Out of 10 cultivated *T*. *equigenitalis* strains, five strains were selected for whole genome sequencing (Table 2). Overall, the positivity rate among the stallions tested was 30.3% and only 13.8% among all samples, including multiple samples per stallion, suggesting that the positive samples were truly positive. All stallion names were encoded (Table 1).

**Table 1:** Samples isolated from Kladruber stallions that tested positive for *T*. *equigenitalis* cultivation.

Stallion	*Fossa glandis*	*Ostium urethrae*	*Praeputium*	Ejaculate	Year/place of isolation
Stallion A	**+**	**+**	-	**+**	2021/Kladruby (CZ)
Stallion B	**+**	-	-	n.t.	2021/Kladruby (CZ)
Stallion C	**+**	-	-	n.t.	2021/Kladruby (CZ)
Stallion D	**+**	-	-	n.t.	2021/Kladruby (CZ)
Stallion E	**+**	-	-	n.t.	2021/Kladruby (CZ)
Stallion F	**+**	**+**	-	n.t.	2022/Kladruby (CZ)
Stallion G	-	-	-	**+**	2022/Kladruby (CZ)
Stallion H	**+**	**+**	-	-	2023/Kladruby (CZ)
Stallion I	**+**	-	-	-	2023/Kladruby (CZ)
Stallion J	**+**	**+**	-	n.t.	2023/Kladruby (CZ)

+, positive cultivation result; -, negative cultivation result; n.t., not tested

**Table 2:** Characteristics of sequenced *T*. *equigenitalis* strains.

Sample name	Original sample name	Year of isolation	Sex	Place of isolation	Genome size (bp)	Average genome coverage		Accession number (GenBank)
						Illumina	Nanopore	
**KLA1**	CAPM 6344	1982	Mare	Kladruby (CZ)	1,668,338	700×	606×	CP155836
**KLA2**	CAPM 6345	1982	Mare	Kladruby (CZ)	1,668,340	671×	546×	CP155829
**KLA3**	Stallion A	2021	Stallion	Kladruby (CZ)	1,667,855	577×	453×	CP155830
**KLA4**	Stallion B	2021	Stallion	Kladruby (CZ)	1,667,854	585×	443×	CP156040
**KLA5**	Stallion C	2021	Stallion	Kladruby (CZ)	1,667,544	611×	304×	CP155831
**KLA6**	Stallion D	2021	Stallion	Kladruby (CZ)	1,667,544	640×	393×	CP155832
**KLA7**	Stallion E	2021	Stallion	Kladruby (CZ)	1,667,544	583×	345×	CP155833
**KYJ**	CAPM 6606	2017	Mare	Kyjov (CZ)	1,668,279	495×	466×	CP155834
**TLU**	CAPM 6629	2018	Mare	Tlumačov (CZ)	1,668,351	606×	466×	CP156890
**UK1**	CCM 6190^T^	1977	Mare	England (UK)	1,731,205	654×	237×	CP155835

### Whole genome sequencing of *T*. *equigenitalis* retrieved from contemporary and historical samples

In total, the complete genome sequences of ten *T*. *equigenitalis* strains were determined and analyzed in this study. The GenBank accession numbers of these sequences are listed in Table 2 and S5 Table. Nine strains originated from Kladruber horses in the Czech Republic, with the exception of the TLU strain, which originated from a different horse breed (Table 2). Five of these were from samples collected in 2021 (Table 1), while the remaining four sequenced strains were reference strains isolated in 1982, 2017, and 2018. Finally, the whole genome sequence of a *T*. *equigenitalis* strain (UK1; CCM6190^T^) originally isolated in the United Kingdom was also obtained by re-sequencing. Sequencing of all isolates resulted in closed whole genomes.

### Antibiotic susceptibility of *T*. *equigenitalis* strains

All sequenced *T*. *equigenitalis* strains were tested for susceptibility to selected antibiotics (Table 3). All strains formed inhibition zones around disks containing gentamicin, nitrofurantoin, and rifampicin, except UK1 with rifampicin. However, none of the isolates were susceptible to sulfamethoxazole/trimethoprim. *T*. *equigenitalis* reference strains isolated between 1982 and 2018 (KLA1, KLA2, KYJ, and TLU) were inhibited by streptomycin (zone diameter >36 mm). Conversely, all contemporary strains (KLA3, KLA4, KLA5, KLA6, and KLA7) and the reference strain UK1 were resistant to streptomycin (Table 3).

**Table 3:** Antibiotic susceptibility of *T*. *equigenitalis* strains and point mutations present in the *rpsL* gene.

Sample name	Original sample name	Zone diameter (millimeters)					Nucleotide present at position 1,630,702 (in *rpsL*)
		CN	F	RD	SXT	S	
**KLA1**	CAPM 6344	> 26	> 47	> 13	6	> 36	T
**KLA2**	CAPM 6345	> 26	> 47	> 13	6	> 36	T
**KLA3**	Stallion A	> 26	> 47	> 13	6	6	C
**KLA4**	Stallion B	> 26	> 47	> 13	6	6	C
**KLA5**	Stallion C	> 26	> 47	> 13	6	6	C
**KLA6**	Stallion D	> 26	> 47	> 13	6	6	C
**KLA7**	Stallion E	> 26	> 47	> 13	6	6	C
**KYJ**	CAPM 6606	> 26	> 47	> 13	6	> 36	T
**TLU**	CAPM 6629	> 26	> 47	> 13	6	> 36	T
**UK1**	CCM6190^T^	> 26	> 47	6	6	6	C

Gentamicin (CN; 10 μg), nitrofurantoin (F; 100 μg), rifampicin (RD; 5 μg), streptomycin (S; 10 μg), sulfamethoxazole/trimethoprim (SXT; 25 μg); each disk measured 6 mm in diameter. The *rpsL* gene was screened for mutations associated with the streptomycin resistance phenotype. A substitution was identified in different strains at position 1,630,702 (coordinate in NZ_CP120814.1).

### Phylogeny of *T*. *equigenitalis* from recent and historical samples

Ten genomes sequenced in this study and a set of available *T*. *equigenitalis* whole genome sequences (n = 18) were used for analyses (listed in S1 Table). Prior to phylogenetic analysis, putatively recombinant genes or genomic loci were removed from the alignments (Materials and methods). In 28 *Taylorella equigenitalis* sequences, an average of 6.6% of the genome was identified as regions potentially containing recombinations (see S1 Fig and S2 Table).

**Fig 1:**
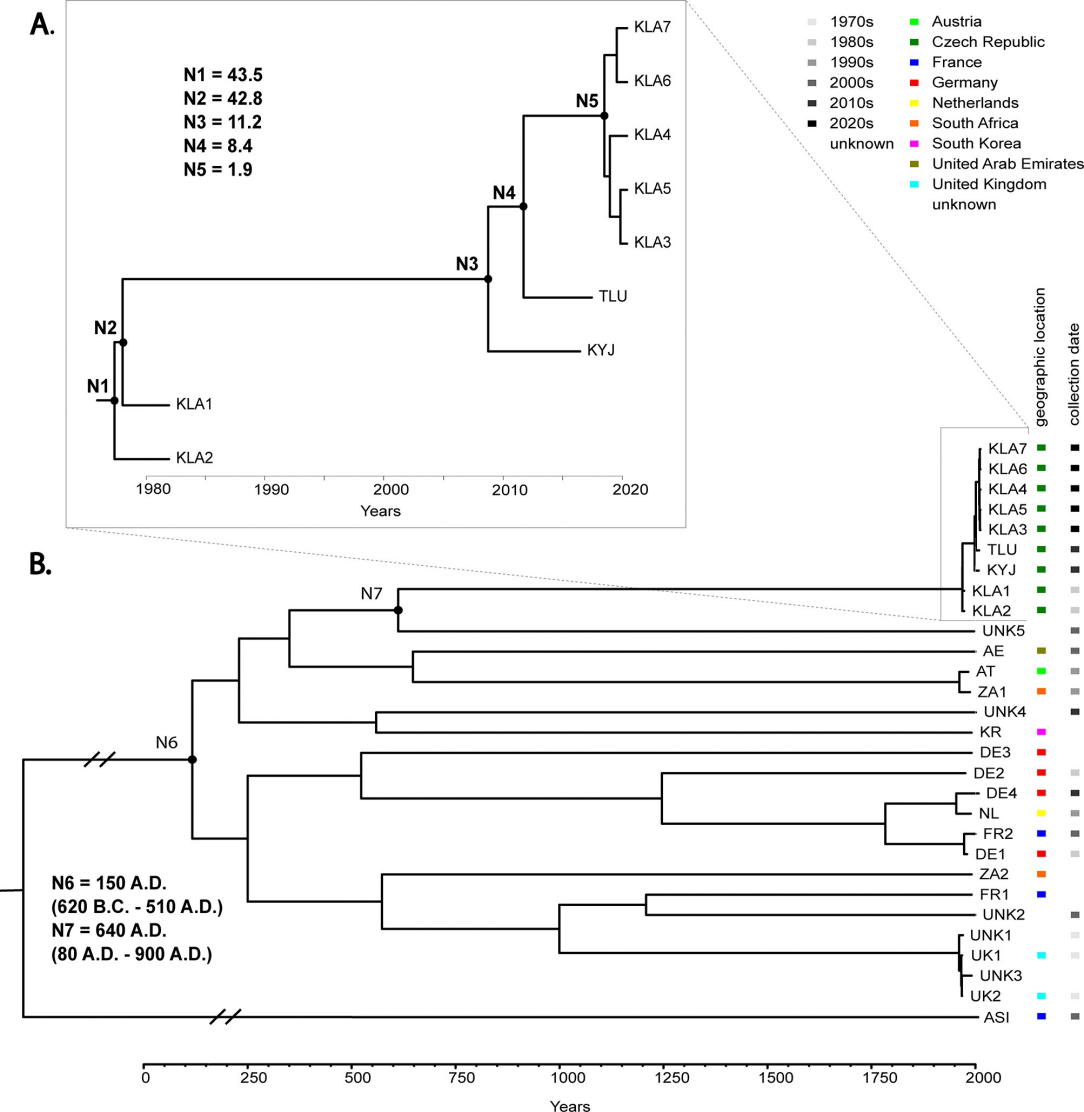
**A.** BEAST-constructed tree of the genome alignments of 9 Czech strains. The divergence date estimates (median and 95% HPD; highest posterior density) for major nodes in the tree are shown. **B.** BEAST-constructed tree of the genome alignments of all 28 samples of *T*. *equigenitalis* genomes. *T*. *asinigenitalis* was used as an outgroup for the phylogenetic tree. The relatively high diversity observed between the different strains is evident as long branches. The nodes describing the most recent common ancestor of individual groups and all genomes are shown. N represents the estimated years of divergence for the strains originating from the node.

The BEAST tree was constructed using the genome alignments of 9 Czech strains (from Kladruby, Kyjov and Tlumačov), all from the same Kladruber horse breed except the TLU strain (Fig 1A). The constructed phylogenetic tree exhibited short branches, indicating a close relationship between the strains. The differences between individual strains collected at the same time (strains KLA3—KLA7) showed an average mean distance of 2 pairwise substitutions (with a maximum of 6). The isolation dates of the Kladruber strains were used to calibrate the coalescent constant population demographic model yielding a scaled mean evolutionary rate of 6.9×10^−7^ substitutions per site per year for the entire genome. Detailed results, including summary statistics of the clockRate estimated from the Czech strains, are shown in S4 Table.

All 28 available *T*. *equigenitalis* genomes were then analyzed with substitution rates fixed at the previously calculated value of 6.9×10^−7^ substitutions per site per year. *T*. *equigenitalis* genomes of Kladruber horses clustered with a sample of unknown origin (UNK5; GenBank acc no. ASM2886859v1) with the most recent common ancestor placed at 640 A.D. (80–900 A.D.) and more distantly with the Austrian sample and the samples from South Africa and the United Arabian Emirates (Fig 1B). The most recent common ancestor of all genomes was placed in the first half of the second century (mean calendar year 150, 95% HPD 620 B.C. - 510 A.D.; see Fig 1B).

## Discussion

The National Stud in Kladruby nad Labem, Czech Republic, is one of the oldest studs in the world with a history of more than 400 years. The Kladruber horses represent the oldest indigenous Czech horse breed and are kept only in one region of the Czech Republic. For centuries, the breeding of Kladruber horses has been restricted to this stud, with no introduction of new horses from outside, suggesting also limited (if any) transfer of horse *T*. *equigenitalis* pathogen strains. Over the recent decades, positive cultivation results for *T*. *equigenitalis* from clinical samples from the Kladruber horses [8, 31, 32] have predominantly been obtained from animals without clinical signs, suggesting that long-term selection of Kladruber horses may have resulted in their increased resistance to *T*. *equigenitalis* infection and/or decreased pathogenicity of local strains of *T*. *equigenitalis*. Swabs from the *fossa glandis* were found to be optimal for cultivation detection of *T*. *equigenitalis*, confirming a previously published finding [33].

Despite this isolation, the Czech *Taylorella* strains from the Kladruber horses clustered together with the Austrian strain, but also with strains from South Africa and the United Arab Emirates. However, the last two are likely samples from exported horses of unknown origin. Similar findings were already obtained using MLST typing of *T*. *equigenitalis*, where Czech samples clustered with German, Swiss and Austrian samples [8], suggesting a common origin of *T*. *equigenitalis* infection in European horse breeds.

Resequencing of the *T*. *equigenitalis* reference strain CCM 6190^T^ (UK1), originally isolated in in England in 1977, revealed four distinct single nucleotide variants compared to the genome sequence provided by the Wellcome Sanger Institute (UK2; assembly number 48853_G02, 2018). Subsequent analysis of the nucleotide variants revealed that the variants observed in our study were well supported by the sequencing reads, indicating that the sequencing error rate in this study is well below 3×10^−6^ and likely even lower. The observed differences in genome sequences likely represent accumulated differences of the *T*. *equigenitalis* CCM6190^T^ strain deposited in different laboratory collections. Independent cultivation prior to collection deposition and also after collection retrieval, together with possible genetic drift, may have contributed to the observed sequence differences.

All sequenced *T*. *equigenitalis* strains from Kladruby clustered tightly together, indicating that the infection has been enzootic in Kladruby for many years and is not newly or repeatedly introduced from outside. This situation allows a straightforward molecular and mutational analyses of the taylorellae, since introduction of strains from outside the stud is highly unlikely. Mutational analysis revealed a scaled mean evolutionary rate of 6.9×10^−7^ substitutions per site per year, which for the entire genome corresponds to 1.13 substitutions per genome and year. While this value is lower than the estimated substitution rate of the human sexually transmitted pathogen *Neisseria gonorrhoeae* (4.54×10^−6^ substitutions per site per year, 6.4 substitutions per genome and year; [34]), it is higher than the substitution rate in another human sexually transmitted pathogen, *Treponema pallidum ssp*. *pallidum* (8.46×10^−8^ substitutions per site per year, less than 0.1 substitutions per genome and year; [35, 36]). The relatively small genome size, low substitution rate, limited horizontal gene transfer, and host specialization of *T*. *equigenitalis* suggest that this bacterium belongs to the group of monomorphic bacteria. Other examples of such bacteria include *Burkholderia mallei*, *Mycobacterium leprae*, *Yersinia pestis*, and *Treponema pallidum* [37, 38]. It is now widely accepted that molecular clock rates differ among bacterial species [39], and that molecular clock rates should be rather determined from short periods of time (years to decades) by analyses within the species rather than from interspecies comparisons [39]. In contrast to short-term mutation rates, long-term mutation rates (calculated over millennia) are much lower (about an order of magnitude), likely as a result of negative (purifying) selection, selection for particular synonymous codons (codon bias), and the effects of genetic drift [39]. The highly controlled breeding of Kladruber horses, with a low risk of introducing new strains of pathogen from external sources, coupled with the clonal nature of *T*. *equigenitalis* strains within the breed, suggests that eradication of the pathogen from the farm could lead to *Taylorella*-free breeding. However, preventive screening and treatment of both breeding horses and potential carrier animals remains crucial.

The Czech *T*. *equigenitalis* strains were similar with respect to their susceptibility to gentamicin, nitrofurantoin and sulfamethoxazole/trimethoprim. However, the UK1 strain was resistant to rifampicin, unlike the other strains tested, likely reflecting its phylogenetic distance from *T*. *equigenitalis* strains from Kladruber horses. Streptomycin susceptibility correlated with the presence of mutations within the *rps*L gene (Table 3). The *rps*L gene encodes the ribosomal protein S12, a well-established mediator of streptomycin resistance in bacteria [40], and the observed mutation (T/C substitution at genomic position 1,630,702; coordinate in NZ_CP120814.1) resulted in the replacement of phenylalanine by serine in the S12 protein. While recent *T*. *equigenitalis* strains from Kladruby and the UK1 strain were found to be resistant to streptomycin, *T*. *equigenitalis* strains from Kladruby isolated before 2018 were streptomycin susceptible, suggesting that this mutation has only recently emerged in the population of *T*. *equigenitalis* from Kladruby. Interestingly, Hicks et al. [4] identified a single point mutation in the *rpsL* gene at residue 43, resulting in a lysine to arginine substitution, as the mechanism of streptomycin resistance in *T*. *equigenitalis*. This finding suggests that additional mutations in this gene may confer further resistance. The newly discovered streptomycin resistance may limit the treatment of *T*. *equigenitalis* as it appears to be widely distributed in the pathogen population.

*T*. *equigenitalis* infecting Kladruber horses clustered with a sample of unknown origin (UNK5; ASM2886859v1) with the most recent common ancestor present around 640 A.D. (80 A.D. - 900 A.D.). Moreover, the most recent common ancestor of all genomes was placed in the first half of the second century (mean calendar year 150, 95% HPD 620 B.C. - 510 A.D.) These estimates suggest that the highest prosperity of the Roman Empire (during the second century A.D.) may have facilitated the spread of *Taylorella* infections across the European continent. At that time, the Roman Empire encompassed extensive territories in Europe, North Africa, and the Near East potentially providing sources of infection. In addition, the Roman centralized control system may have contributed to the spread of infection to various regions. Similarly, as Europe was later divided into smaller territorial units and rebuilt breeding facilities within their territories, *Taylorella* were more likely to evolve independently for an extended period of time. Since the long-term substitution rates (see above) are generally lower than the short-term substitution rates determined in this study, it cannot be excluded that the most recent common ancestor of all genomes dates further into the past, into the time period before Christ. Further molecular dating studies with *Taylorella* isolates from horses from various countries, both within and outside the historical Roman Empire, could test and refine our estimated timing of the common *Taylorella* ancestor.

Several limitations of this work include the relatively limited number of genomes examined from the Czech samples (n = 9), a relatively short sampling period (42 years), and the absence of a randomized or systematic sampling approach, which could result in sampling bias potentially affecting the observed genetic diversity. Moreover, the number of publicly available *T*. *equigenitalis* complete genomes was limited (n = 18) at the time of our analysis. To more clearly analyze the phylogenetic relatedness of *T*. *equigenitalis*, future studies on *T*. *equigenitalis* should be performed on a larger set of genomic data.

Taken together, the genomic analyses of *T*. *equigenitalis* strains in this study suggest the presence of the common ancestor of this pathogen at the time of the greatest extent of the Roman Empire.

## Conclusion

In this study, nine whole genome sequences of *Taylorella equigenitalis* isolated in the Czech Republic between 1982 and 2018 were determined. The collection dates of these strains were used to estimate the substitution rate of *T*. *equigenitalis*, and subsequent analysis with other available *T*. *equigenitalis* genome sequences suggested a common ancestor for *T*. *equigenitalis* strains dating back to the Roman Empire. In addition, a novel mutation conferring streptomycin resistance was identified in recent *T*. *equigenitalis* strains of Czech origin. The limited genetic diversity of *T*. *equigenitalis* strains and the emergence of antibiotic resistance have not only contributed to our understanding of this pathogen, but will also help to prepare new preventive and treatment strategies in the future.

## Supporting information

S1 Table Available complete genomes of *T*. *equigenitalis* used for phylogenetic analyses. (DOCX)

S2 Table Total number of bases spanning putative recombination events identified by Gubbins and their ratio to the genome length. These regions were masked and were not used for phylogenetic analyses.
(DOCX)

S3 Table Evaluation of population models and clock models using nested sampling bayesian computation algorithm in BEAST2. (DOCX)

S4 Table Summary statistics of clockRate estimated from the Kladruber horses. (DOCX)

S5 Table GenBank accession numbers of *T*. *equigenitalis* strains sequenced in this study. (DOCX)

S1 Fig Isolation and PCR-diagnostics of *T*. *equigenitalis* from Kladruber horses. (DOCX)

S2 Fig Predicted recombinant regions in *Taylorella equigenitalis*. (DOCX)
