# Correction: Whole genome sequences of nine *Taylorella equigenitalis* strains isolated in the Czech Republic between 1982–2021: Molecular dating suggests a common ancestor at the time of Roman Empire

**DOI:** 10.1371/journal.pone.0344200

**Published:** 2026-03-02

**Authors:** Matěj Hrala, Petr Andrla, Juraj Bosák, Pavla Fedrová, Amir Mugutdinov, Renata Karpíšková, Kateřina Nedbalcová, Jitka Raichová, Martin Faldyna, Petr Hořín, David Šmajs

In the Discussion section, there is an error in the fourth sentence of the fifth paragraph. The correct sentence is: The rpsL gene encodes the ribosomal protein S12, a well-established mediator of streptomycin resistance in bacteria [40], and the observed mutation (T/C substitution at genomic position 1,630,702; coordinate in NZ_CP120814.1) resulted in the replacement of lysine by arginine in the S12 protein.

In the Discussion section, the last two sentences of the fifth paragraph should not be included.

In the Conclusion section, the third sentence of the paragraph should not be included.
