# Supplementary material for: Whole genome sequences of nine Taylorella equigenitalis strains isolated in the Czech Republic between 1982–2021: Molecular dating suggests a common ancestor at the time of Roman Empire
Source: PLoS One. 2025 Jan 3;20(1):e0315946. doi: 10.1371/journal.pone.0315946 (PMC11698419; doi:10.1371/journal.pone.0315946)
Supplement: S1 Table — (DOCX) [file pone.0315946.s001.docx]

**Supplementary Table 1.** Available complete genomes of *T. equigenitalis* used for phylogenetic analyses

| **Sample name** | **Assembly name** | **Strain designation** | **Collection date** | **Geographic origin** |
| --- | --- | --- | --- | --- |
| **AT** | ASM228802v1 |  | 1992 | Austria |
| **AE** | ASM36724v1 | 14/56 | 2009 | United Arab Emirates |
| **DE1** | ASM228812v1 |  | 1989 | Germany |
| **DE2** | ASM2886801v1 |  | 1985 | Germany |
| **DE3** | ASM2886815v1 |  |  | Germany |
| **DE4** | ASM329389v1 |  | 2017 | Germany: Rhineland-Palatinate |
| **FR1** | ASM2886893v1 | MCE9 |  | France |
| **FR2** | ASM76446v1 |  | 2009 | France: Manche |
| **KR** | ASM2886874v1 |  |  | South Korea |
| **NL** | ASM228824v1 |  | 1998 | Netherlands |
| **UK2** | 48853_G02 | ATCC 35865 | 1977 | United Kingdom: Cambridge |
| **ZA1** | ASM127868v1 |  | 1996 | South Africa: Onderstepoort |
| **ZA2** | ASM2886827v1 |  |  | South Africa |
| **UNK1** | ASM228815v1 |  | 1979 |  |
| **UNK2** | ASM228820v1 |  | 2009 |  |
| **UNK3** | ASM27668v1 | ATCC 35865 |  |  |
| **UNK4** | ASM2886846v1 |  | 2010 |  |
| **UNK5** | ASM2886859v1 |  | 2006 |  |
