# Supplementary material for: Whole genome sequences of nine Taylorella equigenitalis strains isolated in the Czech Republic between 1982–2021: Molecular dating suggests a common ancestor at the time of Roman Empire
Source: PLoS One. 2025 Jan 3;20(1):e0315946. doi: 10.1371/journal.pone.0315946 (PMC11698419; doi:10.1371/journal.pone.0315946)
Supplement: S2 Table — These regions were masked and were not used for phylogenetic analyses. (DOCX) [file pone.0315946.s002.docx]

**Supplementary Table 2.** Total number of bases spanning putative recombination events identified by Gubbins and their ratio to the genome length. These regions were masked and were not used for phylogenetic analyses.

| Sample name | Assembly name | Sequence Length | Cumulative bases in recombinations | Recombination-impacted genome (%) |
| --- | --- | --- | --- | --- |
| UK2 | 48853_G02 | 1732243 | 114073 | 6.6 |
| ZA1 | ASM127868v1 | 1670247 | 80891 | 4.8 |
| AT | ASM228802v1 | 1666291 | 80871 | 4.9 |
| DE1 | ASM228812v1 | 1649945 | 175814 | 10.7 |
| UNK1 | ASM228815v1 | 1739054 | 114073 | 6.6 |
| UNK2 | ASM228820v1 | 1635330 | 54219 | 3.3 |
| NL | ASM228824v1 | 1692042 | 402209 | 23.8 |
| UNK3 | ASM27668v1 | 1732123 | 114073 | 6.6 |
| DE2 | ASM2886801v1 | 1687202 | 137065 | 8.1 |
| DE3 | ASM2886815v1 | 1665615 | 91886 | 5.5 |
| ZA2 | ASM2886827v1 | 1712455 | 17496 | 1.0 |
| UNK4 | ASM2886846v1 | 1729336 | 80802 | 4.7 |
| UNK5 | ASM2886859v1 | 1649350 | 79021 | 4.8 |
| KR | ASM2886874v1 | 1682299 | 98824 | 5.9 |
| FR1 | ASM2886893v1 | 1704267 | 106997 | 6.3 |
| DE4 | ASM329389v1 | 1619593 | 317446 | 19.6 |
| AE | ASM36724v1 | 1652885 | 94638 | 5.7 |
| FR2 | ASM76446v1 | 1668004 | 175820 | 10.5 |
| ASI | ASM22662v1 | 1638559 | 0 | 0.0 |
| KLA1 | CAPM 6344 | 1668044 | 85929 | 5.2 |
| KLA2 | CAPM 6345 | 1668049 | 85929 | 5.2 |
| KLA3 | Stallion E | 1667284 | 85929 | 5.2 |
| KLA4 | Stallion B | 1667272 | 85929 | 5.2 |
| KLA5 | Stallion C | 1667343 | 85929 | 5.2 |
| KLA6 | Stallion A | 1667283 | 85929 | 5.2 |
| KLA7 | Stallion D | 1667333 | 85929 | 5.2 |
| KYJ | CAPM 6606 | 1668018 | 85929 | 5.2 |
| TLU | CAPM 6629 | 1668097 | 85929 | 5.2 |
| UK1 | CCM6190T | 1731481 | 115214 | 6.7F |
