# Supplementary material for: Whole genome sequences of nine Taylorella equigenitalis strains isolated in the Czech Republic between 1982–2021: Molecular dating suggests a common ancestor at the time of Roman Empire
Source: PLoS One. 2025 Jan 3;20(1):e0315946. doi: 10.1371/journal.pone.0315946 (PMC11698419; doi:10.1371/journal.pone.0315946)
Supplement: S3 Table — (DOCX) [file pone.0315946.s003.docx]

**Supplementary Table 3.** Evaluation of population models and clock models using nested sampling bayesian computation algorithm in BEAST2

| Site model | Clock model | Priors | Marginal likelihood | SD |
| --- | --- | --- | --- | --- |
| HKY | strict clock | Coalescent constant population | -2064197 | 8.8 |
| HKY | strict clock | Birth Death model | -2064255 | 12.0 |
| HKY | strict clock | Yule skyline model | -2064235 | 10.9 |
| HKY | optimised relaxed clock | Coalescent constant population | -2064246 | 12.0 |
