# Supplementary material for: Whole genome sequences of nine Taylorella equigenitalis strains isolated in the Czech Republic between 1982–2021: Molecular dating suggests a common ancestor at the time of Roman Empire
Source: PLoS One. 2025 Jan 3;20(1):e0315946. doi: 10.1371/journal.pone.0315946 (PMC11698419; doi:10.1371/journal.pone.0315946)
Supplement: S4 Table — (DOCX) [file pone.0315946.s004.docx]

**Supplementary Table 4.** Summary statistics of clockRate estimated from the Kladruber horses

| mean | 6.8511E-7 |
| --- | --- |
| stderr of mean | 1.5909E-9 |
| stdev | 9.9486E-8 |
| variance | 9.8974E-15 |
| median | 6.785E-7 |
| value range | [3.2905E-7, 1.1112E-6] |
| geometric mean | 6.7789E-7 |
| 95% HPD interval | [4.8564E-7, 8.6941E-7] |

stderr of mean, the standard error of the mean. This takes into account the effective sample size so a small ESS will give a large standard error; stdev, the standard deviation of the samples; geometric mean, the central tendency or typical value of the set of samples; 95% HPD Interval, the lower bound and upper bound of the highest posterior density (HPD) interval. The HPD is the shortest interval that contains 95% of the sampled values.
