# Supplementary material for: Whole genome sequences of nine Taylorella equigenitalis strains isolated in the Czech Republic between 1982–2021: Molecular dating suggests a common ancestor at the time of Roman Empire
Source: PLoS One. 2025 Jan 3;20(1):e0315946. doi: 10.1371/journal.pone.0315946 (PMC11698419; doi:10.1371/journal.pone.0315946)
Supplement: S5 Table — (DOCX) [file pone.0315946.s005.docx]

|  | **Genome sequence** | **BioProject** | **BioSample** | **SRA** |
| --- | --- | --- | --- | --- |
| **KLA1** | CP155836 | PRJNA1110976 | SAMN41379338 | SRR31367430 |
| **KLA2** | CP155829 | PRJNA1111292 | SAMN41387432 | SRR31367471 |
| **KLA3** | CP155830 | PRJNA1111304 | SAMN41387892 | SRR31370644 |
| **KLA4** | CP156040 | PRJNA1111331 | SAMN41388509 | SRR31370300 |
| **KLA5** | CP155831 | PRJNA1111353 | SAMN41388962 | SRR31370457 |
| **KLA6** | CP155832 | PRJNA1111378 | SAMN41390265 | SRR31370226 |
| **KLA7** | CP155833 | PRJNA1111394 | SAMN41390443 | SRR31370592 |
| **KYJ** | CP155834 | PRJNA1111417 | SAMN41390821 | SRR31370178 |
| **TLU** | CP156890 | PRJNA1111418 | SAMN41391237 | SRR31367840 |
| **UK1** | CP155835 | PRJNA1111427 | SAMN41391404 | SRR31362389 |

**Supplementary Table 5:** GenBank accession numbers of *T. equigenitalis* strains sequenced in this study
