# Supplementary material for: Whole genome sequences of nine Taylorella equigenitalis strains isolated in the Czech Republic between 1982–2021: Molecular dating suggests a common ancestor at the time of Roman Empire
Source: PLoS One. 2025 Jan 3;20(1):e0315946. doi: 10.1371/journal.pone.0315946 (PMC11698419; doi:10.1371/journal.pone.0315946)
Supplement: S1 Fig — (DOCX) [file pone.0315946.s006.docx]

**Supplementary Figure 1:** Isolation and PCR-diagnostics of *T. equigenitalis* from Kladruber horses

**A)** *T. equigenitalis* growth on chocolate agar


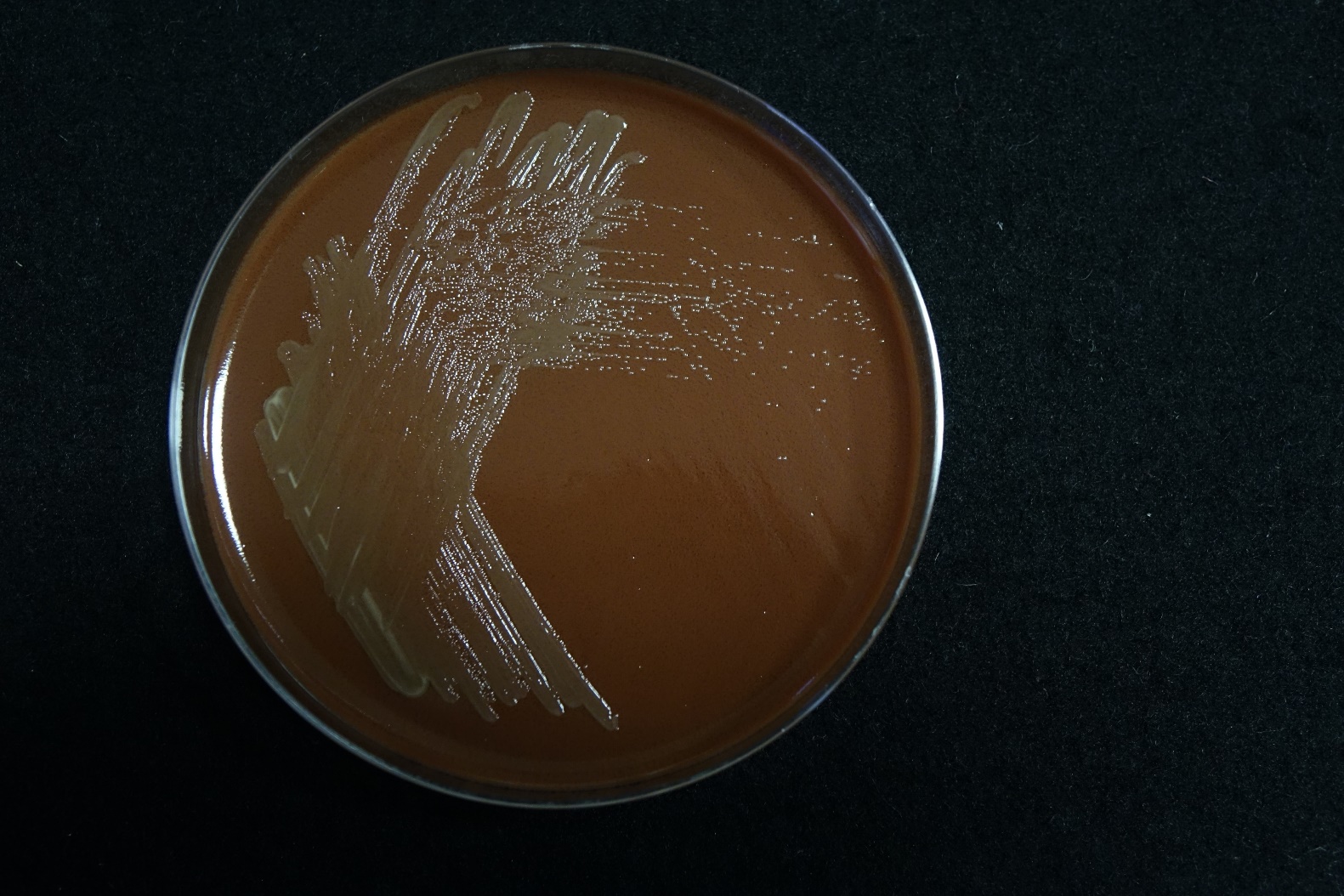


Note: Cultivation of a pure culture of *T. equigenitalis* from Kladruber stallion A.

**B)** Amplification of *gltA* and *txn* loci for *T. equigenitalis* detection (Duquesne *et al.* 2013)


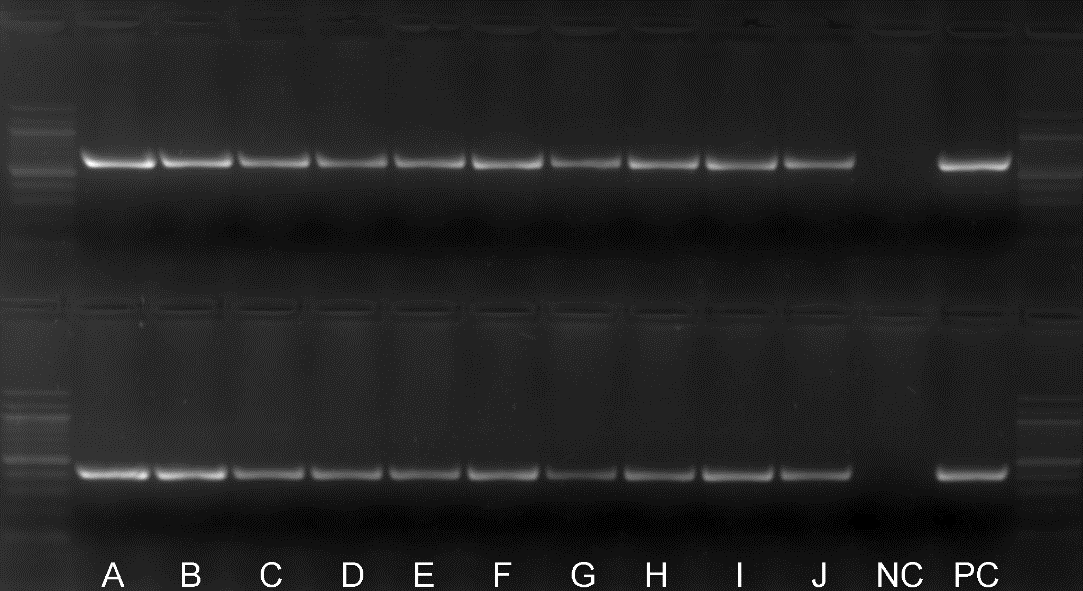


Notes: A total of 10 Kladruber stallions (A-J) tested positive for T. equigenitalis. Suspect colonies were amplified using *Taq* polymerase for the gltA (upper row; 682 bp) and txn (lower row; 455 bp) loci. NC = negative control with no added DNA; PC = positive control with reference strain CCM 6190^T^ (UK1); 100 bp marker was used.

**C)** Sanger sequencing codes (*gltA*) of individual PCR products

| **Code** | **Stallion** |
| --- | --- |
| ETC568 | A |
| ETC565 | B |
| ETC566 | C |
| ETC564 | D |
| ETC567 | E |
| FLN145 | F |
| FLN142 | G |
| IKO888 | H |
| IKO886 | I |
| IKO890 | J |

Note: Reads are available upon request.
