# Supplementary material for: Whole genome sequences of nine Taylorella equigenitalis strains isolated in the Czech Republic between 1982–2021: Molecular dating suggests a common ancestor at the time of Roman Empire
Source: PLoS One. 2025 Jan 3;20(1):e0315946. doi: 10.1371/journal.pone.0315946 (PMC11698419; doi:10.1371/journal.pone.0315946)

**Supplementary Figure 2.** Predicted recombinant regions in *Taylorella equigenitalis*, predicted by the Gubbins software based on full-genome alignment of 28 *Taylorella equigenitalis* isolates aligned against reference genome (GenBank GCA_028868935.1). Recombinant regions which are present across isolates are shown in red, while recombinant regions unique to a single isolate are shown in blue.


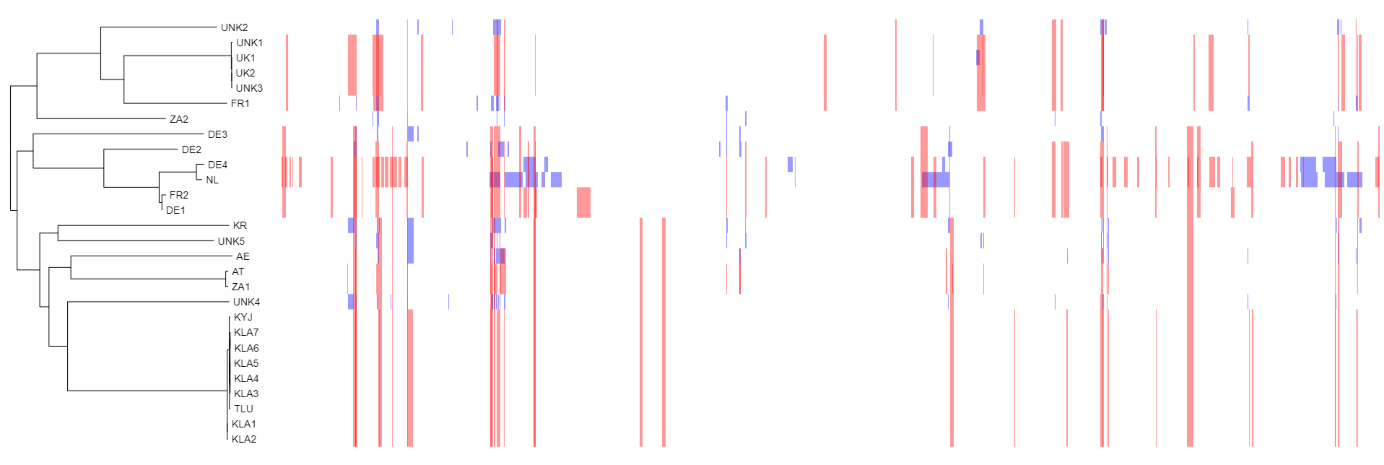

Supplement: S2 Fig — (DOCX) [file pone.0315946.s007.docx]
